# Spanish Validation of the PICCOLO (*Parenting Interactions With Children: Checklist of Observations Linked to Outcomes*)

**DOI:** 10.3389/fpsyg.2019.00680

**Published:** 2019-03-27

**Authors:** Rosa Vilaseca, Magda Rivero, Rosa M. Bersabé, Esperanza Navarro-Pardo, Maria Jose Cantero, Fina Ferrer, Clara Valls Vidal, Mark S. Innocenti, Lori Roggman

**Affiliations:** ^1^Department of Cognition, Development and Educational Psychology, University of Barcelona, Barcelona, Spain; ^2^Department of Psychobiology and Methodology of the Behavioral Sciences, University of Málaga, Málaga, Spain; ^3^Department of Developmental and Educational Psychology, Universitat de València, Valencia, Spain; ^4^Department of Psychology, Universitat Abat Oliba-CEU, Barcelona, Spain; ^5^Center for Persons with Disabilities, Utah State University, Logan, UT, United States

**Keywords:** PICCOLO, Spanish version, parenting interactions, observational scale, psychometric properties

## Abstract

**Background/Objective:** The aim of this study was to explore the psychometric properties of the Spanish version of the *Parenting Interactions with Children: Checklist of Observations Linked to Outcomes* (PICCOLO; Roggman et al., 2013a). This observational measure is composed of 29 items that assess the quality of four domains of parenting interactions that promote child development: affection, responsiveness, encouragement, and teaching.

**Methods:** The sample included 203 mother-child dyads who had been video-recorded playing together. Fifty-six percent of the children were male, and 44% were female, aged from 10 to 47 months. Video-recorded observations were rated using PICCOLO items.

**Results:** Confirmatory factor analysis supported that the instrument has four first-order factors corresponding to the hypothesized domains of parenting behaviors, and a second-order factor corresponding to a general factor of positive parenting. Construct validation evidence was compiled by examining the relationship between PICCOLO scores and child age. As expected, teaching domain and total PICCOLO scores were positively correlated with child age. The Spanish PICCOLO also demonstrated good inter-rater reliability (ranging from 0.69 to 0.84) and internal consistency reliability (ranging from 0.59 to 0.88) for the four domain scores and the total parenting score. Concurrent criterion-related validity was examined via correlations between parenting scores and child cognitive, language and motor skills outcomes, measured using the Bayley Scales of Infant Development.

**Conclusion:** The Spanish version of the PICCOLO meets the criteria for a reliable and valid observational measurement of parenting interactions with children. The psychometric properties of the instrument make it appropriate for general research purposes, but also for program evaluation of Early Intervention and other parenting-support interventions. This measure, focused on parent strengths, could be used to facilitate family-centered practices in early intervention and other programs that have parenting as an outcome.

## Introduction

Early positive parent-child interactions are important for a child’s positive development in both normally developing children (Love et al., 2005; Blair et al., 2014; Vargas-Rubilar and Arán-Filippetti, 2014) and those with developmental delay and disabilities (Spiker et al., 2002; Innocenti et al., 2013). Early positive parenting interactions promote child development, and specific parenting behaviors that lead to better developmental outcomes have been identified (Bradley and Corwyn, 2002; Mahoney, 2009; Warren et al., 2010). Those parental behaviors that promote child development, primarily studied in mothers but also in fathers in some cases (Anderson et al., 2013; Cabrera et al., 2017), are labeled *parenting* or *positive parenting*. Although we use the term *parenting*, this refers to the child’s caregiver, which may or may not be the parent.

Specific aspects of early parenting have been related to different developmental outcomes in children. On the one hand, parents’ emotional warmth and affection have been related to the child’s social emotional development and secure attachment (Laible et al., 2000; Zhou et al., 2002; Caspi et al., 2004; Sanders et al., 2004). On the other hand, responsive behaviors have also been linked to children’s developmental outcomes. Parental responsive behaviors are quick and contingent responses to the child, adjusted to his/her initiative and interests. Responsiveness has been linked to emotional, social, cognitive and linguistic development (Landry et al., 2001, 2006; Crouter and Head, 2002; Davidov and Grusec, 2006; Hirsh-Pasek and Burchinal, 2006; Bernier et al., 2010; Roseberry et al., 2013; Tamis-LeMonda et al., 2001, 2014). Further, parental behaviors that promote some degree of autonomy, on the part of the child, and that are adjusted to the child’s competencies, setting limits and demanding maturity according to age, have been associated with social and cognitive development and readiness for school (Hubbs-Tait et al., 2002; Joussemet et al., 2005; Bernier et al., 2010, 2012; Graziano et al., 2011; Fay-Stammbach et al., 2014). Finally, cognitive and linguistic stimulation (e.g., explanations, asking the child questions, promoting the child’s participation in adult-child joint activities and conversation) have also been related to children’s cognitive, linguistic, and social emotional development as well as to their emergent literacy skills (Tamis-LeMonda et al., 2001; Hubbs-Tait et al., 2002; Kim-Cohen et al., 2004; Bingham, 2007; Farah et al., 2008).

Different tools have been used to measure the quality of parent-child interactions or positive parenting, most relying on parent reports. Typically, parent reports address parental abilities or competences in a broad sense, and parents respond to different items in terms of their consciousness about their own most frequent behaviors and attitudes about their child’s rearing and education. Good examples of this kind of instrument are the Alabama Parenting Questionnaire (Essau et al., 2006), the Baby Care Questionnaire (Winstanley and Gattis, 2013), and those developed by Gómez and Muñoz (2014) in Chile, and by Vázquez et al. (2016) in Spain. Although these tools can be useful for gaining knowledge about parental competences, they have some limitations that are common to all measures based on self-reporting, which include limitations on validity. When filling out self-report questionnaires, different parents can interpret items differently, not remember well, want to give a good image of themselves… And, even though well-administered questionnaires can offer valuable and useful information, the direct observation of interaction processes between parents and children offers relevant and complementary information.

Some authors have pointed out that direct observation of parental interactions by qualified coders can provide more accurate data about parenting in face-to-face daily interactions (Roggman et al., 2013b). The HOME (Home Measure of the Environment; Caldwell and Bradley, 2003), probably the most broadly used instrument for assessing the quality of family as a developmental context, includes some direct observations of parent-child interactions but is primarily conducted through interview. In Spain, Trenado and Cerezo (2007) developed a sequential coding system of early caregiver-child interaction in real time (CITMI-R). This coding system provides microanalytic measurements of the early dyadic interaction in a free play situation and has adequate reliability in Spanish and English samples (Trenado et al., 2014). Also, in Spain, Velasco et al. (2014) developed another family context assessment instrument (Etxadi-Gangoiti scale), which also includes some items of direct observation of parenting interactions. Although these direct observation measures tend to have good reliability and validity they are time intensive to code and few have been used cross-culturally. For more information about these tools see the review by Alcantud et al. (2015).

Parenting interaction characteristics depend on both personal and cultural factors. Parenting practices are diverse among cultures and reflect cultural child rearing expectations as well as cultural beliefs and values about child development. This does not mean that parenting is intergenerationally transmitted in an automatic way. Every parent conducts his/her parenting behaviors according to personal characteristics and beliefs, individual experience, cultural background and contextual demands. Although cross-cultural studies have been conducted (e.g., Jackson-Newsom et al., 2008; Burchinal et al., 2010; Bornstein, 2013; Lansford et al., 2014), these studies do not typically use a common measure across cultural groups. To better understand the global context of parenting, we need a single tool that could be used among different countries and cultural populations. A tool that is based on direct observation and has potential for cross-cultural use is the PICCOLO (Roggman et al., 2013b). PICCOLO was developed following an in-depth review of previous literature about parental behaviors related to children’s developmental outcomes and the authors’ own studies, and it is composed of 29 items in four behavioral domains. These domains are called affection, responsiveness, encouragement and teaching (Roggman et al., 2013a). As mentioned above, emotional warmth and affection (affection), responsiveness or responsivity (responsiveness), parental control and promotion of autonomy (encouragement), and cognitive and linguistic stimulation (teaching) are the main dimensions of parental behaviors during parent-child interactions that literature has related to the optimization of child development. Each domain identifies specific kinds of developmentally supportive parenting practices that predict children’s outcomes (Innocenti et al., 2013; Roggman et al., 2013a).

The PICCOLO has been shown to have strong reliability and validity in multiple ethnic groups in the United States (788 European American, 792 African American, and 468 Latino American families) and appears applicable across the developmental spectrum from 10 to 47 months. The PICCOLO has been translated into many languages using a process that includes back translation while working with the authors to ensure content validity (Innocenti and Roggman, 2018). This procedure has been used for translations into Spanish for use in Chile and Spain, and into German, Chinese, Italian, Turkish, Brazilian-Portuguese and Dutch (personal communication with the PICCOLO authors). However, only two studies have analyzed psychometric properties of PICCOLO adaptations to other cultures: The Turkish validation of the PICCOLO with a sample of 130 mother-child dyads (Bayoğlu et al., 2013), and the Brazilian-Portuguese validation with a sample of 156 mother-child (18-month-old) dyads (Schneider, 2018).

In Spain, a tool for assessing parenting both for research purposes (e.g., to gain general knowledge about parent-child interactions and their association with developmental outcomes, or to conduct intercultural comparisons about parenting) and for applied purposes in early intervention programs (e.g., to identify parenting strengths as part of an intervention) is needed. It should be an easy-to-administer and easy-to-score observational tool that can provide accurate data about parent-child interactions, be sensitive to changes in response to intervention, and be useful for practitioners who aim to improve parental interactions and thus child development (Gardner, 2000; Aspland and Gardner, 2003; Roggman et al., 2013b; Zaslow et al., 2006). The Spanish validation of the PICCOLO is a contribution in this regard, as the PICCOLO appears to meet these requirements. Unlike other tools mentioned above, the PICCOLO requires little administration time (10 min of audiovisual recording and 20–30 min of coding), and its international use in different ethnic backgrounds opens the way to sharing results between researchers and carrying out intercultural studies.

The purpose of this study was to test the psychometric properties of the PICCOLO in a large sample of Spanish mothers and children aged from 10 to 47 months, in order to validate the tool for use in the Spanish population, for research or applied purposes.

## Materials and Methods

### Instruments

The *Parenting Interactions with Children: Checklist of Observations Linked to Outcomes* (PICCOLO; Roggman et al., 2013a) is a checklist of 29 observable behaviors used to assess parenting interactions with children in four domains: affection, responsiveness, encouragement, and teaching. This instrument is a reliable and valid measure of parent-child interactions for parents with children between the ages of 10 and 47 months. The PICCOLO was developed in the United States based on a sample of over 2,048 low-income families from diverse ethnic groups with at least one child between 10 and 47 months of age, from the archive of the Early Head Start Research and Evaluation Project (Roggman et al., 2013a). Parent-child dyads were observed during 10 min of free play using three bags containing, respectively, a book, toys for pretend play and manipulative toys (Fuligni and Brooks-Gunn, 2013). The 29 items reflect parent interaction behaviors and are scored according to their frequency as 0 (absent, no behavior observed), 1 (barely, minor or emerging behavior) and 2 (clearly, definitive, strong and frequent behavior). They are grouped into four dimensions: (a) Affection (seven items), which involves physical and verbal expression of affection, positive emotions, positive evaluation and positive regard; (b) Responsiveness (seven items), which includes reacting sensitively to a child’s cues and expressions of needs or interests and reacting positively to the child’s behavior; (c) Encouragement (seven items), which considers parents’ support of children’s efforts, exploration, independence, play, choices, creativity, and initiative; and (d) Teaching (eight items), which includes cognitive stimulation, explanations, conversation, joint attention, and shared play. The instrument generates a score for each dimension between 0 and 14 (and 0 to 16 for teaching dimension) and a total score between 0 and 58 (by summing all the items).

The original PICCOLO reliability is good, with an average of 0.77 inter-rater reliability correlations between pairs of observers for total score (0.80 for affection, 0.76 for responsiveness, 0.73 for encouragement and 0.69 for teaching). The correlation of total score between observers of different ethnicities averaged 0.80 (0.78 for affection, 0.68 for responsiveness, 0.66 for encouragement and 0.75 for teaching). The analysis of Cronbach’s Alpha for the total instrument was 0.91 (0.78 for affection, 0.75 for responsiveness, 0.77 for encouragement and 0.80 for teaching) and the instrument had good results for construct and predictive validity (Roggman et al., 2013a). The PICCOLO has also been used to observe parents interacting with children with a disability, showing both reliability and validity (Innocenti et al., 2013).

Child development was assessed using the Spanish version of the *Bayley Scales of Infant Development - III* (BSID-III; Bayley, 2015). BSID-III scales are widely used to assess development between 1 and 42 months of age. Cognitive, Expressive Language, Receptive Language, Fine Motor, and Gross Motor Subscales were applied. While not used in the present study, the BSID-III also includes a Social-Emotional Scale and Adaptive Behavior Scale, which are questionnaires for caregivers. Cognitive, motor and linguistic percentiles were calculated. The Bayley Scales are standardized and have good inter-rater reliability and are valid for predicting current and future development.

### Translation and Adaptation

Consent was obtained from the authors of the original PICCOLO (Roggman et al., 2013a) and publisher (Brookes Publishing) to develop the Spanish version. Two native speakers of Spanish, both experts in Developmental Psychology, translated the original scale from English into Spanish. An English native speaker back-translated it into English. The back-translated form was evaluated by two of the PICCOLO authors (Roggman and Innocenti), who provided suggestions for refinements. The final adapted Spanish version was produced after incorporating their suggestions.

### Participants

Participants were recruited from pediatric centers, nurseries and Community Family Centers. The following criteria were used for inclusion of children in the study: (a) child’s age between 10 and 47 months; (b) child’s birth weight of 2.5 kg (5 pounds 8 ounces) or more; (c) not having complications in childbirth; and (d) no hospitalizations prior to enrollment in the study. Written informed consent was obtained from the participants of this study and from the parents/legal guardians of all non-adult participants.

The Spanish sample included 203 mother-child dyads who had been video recorded playing together. Fifty-six percent of the children were male, and 44% were female, aged from 10 to 47 months (*M* = 27.1, *SD* = 9.8). Three percent of children were less than 1 year old (10 to 11 months), 38% were 1 year old (12 to 23 months), 36% were 2 years old (24 to 35 months), and 23% were 3 years old (36 to 47 months). The mothers were aged between 22 and 47 years (*M* = 34.6, *SD* = 4.3). The majority of mothers were married or living with a partner (95.1%). Most of them (69.1%) had a university degree, 24.1% had completed high school, and 6.8% had received only elementary schooling. They were either full (59.5%) or partially employed (27.2%), or cared for their children and were fully responsible for housework (13.3%). Almost a third of the sample (29%) had a monthly family net income between €1,313 and €2,451, considered an average income in Spain. The monthly income was lower than €1,313 for 6% of the families, and higher than €2,451 for the rest (65%).

### Training of the Raters

A university research group scored the Spanish version of the PICCOLO. All scorers were psychologists and specialists in child development. The first author, who was trained by the authors of the original PICCOLO, trained the raters for this study. Observer trainees read about the content and purpose of the measure (during a 3-h session) and watched and discussed four video recordings (3 h). At the end of the training sessions, the observers watched and coded four to six additional video-recorded interactions to establish reliability (3–6 h). The observers were considered to have completed their training satisfactorily when the percentage inter-rater agreement was equal to or above 80%.

### Procedure

Initially, ethical approval was obtained from the University of Barcelona’s Bioethics Commission (CBUB), according to the International Ethical Guidelines for Health-related Research Involving Humans prepared by the Council for International Organizations of Medical Sciences (CIOMS) in collaboration with the World Health Organization (WHO), and the WMA Declaration of Helsinki - Ethical Principles for Medical Research Involving Human Subjects.

Later on, pediatric centers, nurseries and Community Family Centers were contacted by letter and telephone and informed of the study. We contacted the coordinators of the centers to request their collaboration in recruiting families for the study. Families were informed that their participation would be entirely voluntary and anonymous. Families were mailed questionnaire packages containing a newsletter with information about the study and a brief guide about how to video record at home, an informed consent to sign, and a demographic questionnaire to complete. Mothers were asked to record a video of an approximately 10-minute (between 8 and 10 min) play session with their child at home, with the following instruction: “Interact and play with your children as you typically do.” Mothers recorded themselves and then they sent the video tape to us by mail or we collected them. The videos used in this research were those in which the parent followed the directions of the researchers. The most frequent toys that mothers and children used to play together were books, toy animals, kitchens, little dolls, building blocks… So, the selected toys were very similar to those used by Roggman et al. (2013b) in their original study. Twelve video recordings had to be excluded, either because only the child appeared on the tape, or the audio was not clear enough or because they were shorter than 8 min. The PICCOLO was then used to score parent-child interactions from these approximately 10-minute-long video recordings.

To test criterion-related validity, a subsample of 64 children were randomly selected and were assessed using the cognitive, motor and linguistic subscales of the Bayley Scales of Infant Development (BSID-III; Bayley, 2015). The smaller number of participants for this subsample was due to the high cost of applying the Bayley Scales to the children. The PICCOLO domain scores for each observation were correlated with the Bayley scores.

### Data Analysis

According to the original version of the PICCOLO, video observations rated independently by two coders (*N* = 61) were used to estimate inter-rater reliability via percentage observer agreement for each item, and intraclass correlation coefficients (ICCs) for each domain and total scores.

Video observations rated by one of the two coders (*N* = 203) were used to analyze the different psychometric properties of the instrument. Cronbach’s α coefficient was estimated to determine the internal consistency reliability of the instrument. For element analyses, we calculated Cronbach’s alpha if an item was deleted, and discrimination indexes, obtained as the corrected correlation of the item score with that of the corresponding domain.

Dimensionality of the instrument was examined through confirmatory factor analysis. Since items were rated on a 3-point ordinal scale, diagonally weighted least squares (DWLS) were used to estimate the model parameters. DWLS is considered a robust estimator for ordinal data and small samples and in cases of violations of normality (Forero et al., 2009). Goodness of fit of the model was evaluated according to the following criteria: (a) relative chi-square (χ^2^/df): a good fit is indicated by a value lower than 2; (b) comparative fit index (CFI) and Tucker-Lewis index (TLI): a ≥ 0.90 value indicates an acceptable fit, while a ≥ 0.95 value is an indicator of a good fit; and (c) root mean square error of approximation (RMSEA): a value of ≤ 0.08 RMSEA is indicative of an acceptable fit, while a value of ≤ 0.05 RMSEA is indicative of a good fit (Hu and Bentler, 1999).

Pearson’s product-moment correlation coefficient was used to analyze the relationship between age (in months) and PICCOLO scores. For independent samples, the Student’s *t* test was used to compare mean Spanish PICCOLO scores according to child gender. Discriminant validity was analyzed by obtaining the Pearson correlation coefficients among different PICCOLO domains.

Finally, concurrent criterion-related validity was examined (for a subsample of 64 participants) via Pearson’s correlations between mothers’ PICCOLO scores and children’s scores on the Bayley scales of infant development. Using a two-sided test, with 5% significance and a power of 80%, the required sample size to detect a correlation ≥ 0.32 is approximately 74.

Confirmatory factor analysis was performed using lavaan, an *R* package for Structural Equation Modeling, version 0.5–12 (Rossel, 2012). The rest of the statistical analyses were carried out using IBM SPSS Statistics, version 24.0.

## Results

### Reliability

Video observations rated independently by two coders (*N* = 61, 30,05%) were used to estimate inter-rater reliability. Item-level observer agreement ranged from 54 to 95%. Only item 5 in the encouragement domain (*Verbally encourages child’s efforts*) showed a low inter-rater agreement (<60%). Averaging across items, agreement was 83% for the affection domain, 83% for the responsiveness domain, 78% for the encouragement domain, 77% for the teaching domain, and 80% for all the items of the Spanish PICCOLO. Inter-rater reliability was also estimated via ICCs for each domain and total scores. ICCs were 0.83 for the affection domain, 0.69 for the responsiveness domain, 0.81 for the encouragement domain, 0.80 for the teaching domain, and 0.84 for the total Spanish PICCOLO score. These results demonstrate that there was high agreement between the scores given to each item by the two observers and, consequently, domain and total scores also showed high inter-rater reliability.

Video observations rated by one of the two coders (*N* = 203) were used to analyze the scale’s internal consistency reliability. Internal consistency of the scale was indicated by a Cronbach’s α coefficient of 0.59 for the affection domain, 0.75 for the responsiveness domain, 0.79 for the encouragement domain, 0.68 for the teaching domain, and 0.88 for the total Spanish PICCOLO score. All domain and total scores showed a satisfactory alpha coefficient exceeding an acceptable minimum of 0.65 (DeVellis, 2016), except the alpha coefficient for the affection domain. While a value of 0.65 is generally agreed to be an acceptable value, some researchers (e.g., Cho and Kim, 2015) caution against applying any arbitrary or automatic cutoff criteria. Rather, it is suggested than any minimum value should be determined on an individual basis based on the purpose of the research, the number of items in the scale, and/or the stage of research (i.e., exploratory, basic, or applied). The alpha coefficient for the affection domain was very close to 0.60, which may be considered as acceptable given that the score is composed of only seven items. For item analyses, we calculated Cronbach’s alpha if an item was deleted, and discrimination indexes, obtained as the corrected correlation of the item score with that of the corresponding domain (Table 1). Except for two items, the alpha coefficient of each domain decreased if any of the items was deleted, indicating that all of them contribute to increasing the domain’s internal consistency. In addition, all items (except two) showed discrimination indexes above the recommended minimum of 0.30 (DeVellis, 2016). Item 5 in the affection domain *(Uses positive expressions with child)*, and item 6 in the teaching domain *(Does activities in a sequence of steps)* did not contribute to the α scale, and their discrimination index was below the recommended minimum of 0.30. Indeed, as shown in Table 1, if item 5 was deleted from the affection domain, the alpha coefficient would be 0.65 instead of 0.59, and if item 6 was deleted from the teaching domain, the alpha coefficient would be 0.71 instead of 0.68.

**Table 1:** Item analysis of the Spanish version of the PICCOLO (*N* = 203) (both in English and Spanish).

Domains and Items	*M*	*SD*	Corrected item	Cronbach’s α if
			total correlation	deleted item
Affection (domain scale α)				(0.59)
1. Habla con un tono de voz cariñoso	1.87	0.34	0.38	0.54
[Speaks in a warm tone of voice]				
2. Sonríe al niño/a	1.58	0.58	0.37	0.52
[Smiles at child]				
3. Elogia al niño/a	1.59	0.65	0.34	0.54
[Praises child]				
4. Está físicamente cerca del niño/a	1.93	0.26	0.36	0.56
[Is physically close to child]				
5. Utiliza expresiones positivas con el niño/a	0.73	0.85	0.18	0.65
[Uses positive expressions with child]				
6. Se implica plenamente con el niño/a en la interacción	1.90	0.32	0.30	0.56
[Is engaged in interacting with child]				
7. Muestra calidez emocional	1.70	0.51	0.50	0.48
[Shows emotional warmth]				
Responsiveness (domain scale α)				(0.75)
1. Presta atención a lo que hace el niño/a	1.89	0.33	0.43	0.73
[Pays attention to what child is doing]				
2. Cambia el ritmo o la actividad para ajustarse a los intereses o las necesidades del niño/a	1.62	0.60	0.51	0.71
[Changes pace or activity to meet child’s interests or needs]				
3. Es flexible ante el cambio de actividades o intereses del niño/a	1.63	0.62	0.50	0.72
[Is flexible about child’s change of activities or interests]				
4. Sigue de cerca lo que el niño/a intenta hacer	1.82	0.42	0.56	0.71
[Follows what child is trying to do]				
5. Reacciona ante las emociones del niño/a	1.67	0.54	0.49	0.71
[Responds to child’s emotions]				
6. Mira al niño/a cuando éste habla o emite sonidos	1.79	0.45	0.42	0.73
[Looks at child when child talks or makes sounds]				
7. Responde a las palabras o los sonidos del niño/a	1.72	0.56	0.43	0.73
[Replies to child’s words or sounds]				
Encouragement (domain scale α)				(0.79)
1. Espera la respuesta del niño/a tras hacer una sugerencia	1.59	0.60	0.52	0.75
[Waits for child’s response after making a suggestion]				
2. Anima al niño/a a manipular juguetes	1.82	0.43	0.52	0.76
[Encourages child to handle toys]				
3. Apoya al niño/a para que tome la iniciativa	1.55	0.63	0.51	0.75
[Supports child in making choices]				
4. Apoya al niño/a cuando hace cosas por sí mismo	1.56	0.57	0.59	0.74
[Supports child in doing things on his/her own]				
5. Anima verbalmente los esfuerzos del niño/a	1.33	0.75	0.51	0.76
[Verbally encourages child’s efforts]				
6. Ofrece sugerencias para ayudar al niño/a	1.44	0.66	0.48	0.76
[Offers suggestions to help child]				
7. Muestra entusiasmo acerca de lo que está haciendo el niño/a [Shows enthusiasm about what child is doing]	1.67	0.53	0.48	0.76
Teaching (domain scale α)				(0.68)
1. Explica al niño/a las razones acerca de algo	1.00	0.87	0.54	0.60
[Explains reasons for something to child]				
2. Sugiere actividades para ampliar lo que el niño/a está haciendo [Suggests activities to extend what child is doing]	1.58	0.64	0.48	0.62
3. Repite o expande las palabras o los sonidos del niño/a	1.62	0.67	0.43	0.63
[Repeats or expands child’s words or sounds]				
4. Da nombre a objetos o acciones	1.76	0.51	0.41	0.64
[Labels objects or actions for child]				
5. Participa en el juego simbólico o de ficción del niño/a	1.14	0.91	0.30	0.67
[Engages in pretend play with child]				
6. Realiza las actividades en una secuencia de pasos	1.21	0.90	0.14	0.71
[Does activities in a sequence of steps]				
7. Habla al niño/a acerca de las características de los objetos	1.32	0.78	0.42	0.63
[Talks to child about characteristics of objects]				
8. Pide información al niño/a	1.65	0.61	0.37	0.65
[Asks child for information]				

### Factor Analysis

**Figure 1:**
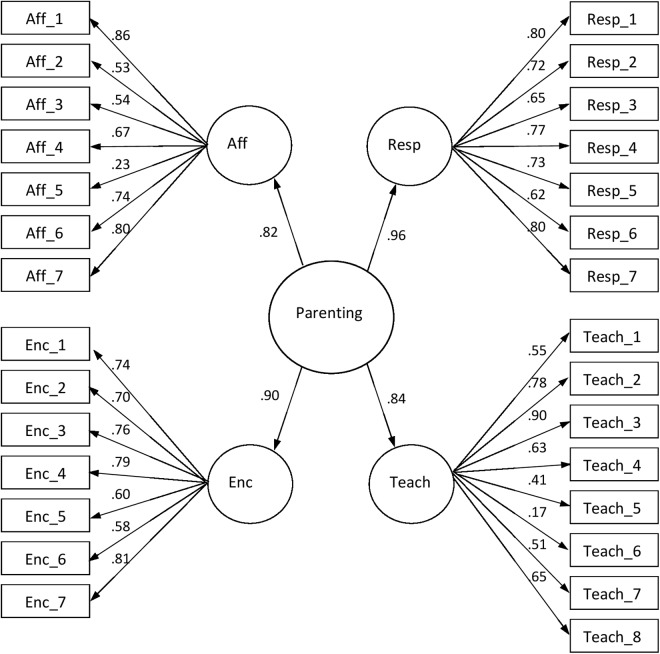
Path diagram of the confirmatory factor analysis. Aff, Affection domain; Resp, Responsiveness domain; Enc, Encouragement domain; Teach, Teaching domain. Affl indicates Item 1 in the affection domain; this shorthand is used throughout. Error terms are omitted.

Because the Spanish PICCOLO was developed using a model based on the original PICCOLO domains (Roggman et al., 2013a), the dimensionality of the instrument was examined using confirmatory factor analysis. We tested a model with four first-order factors corresponding to the four hypothesized domains of parenting behaviors (affection, responsiveness, encouragement, and teaching), and a second-order factor corresponding to a general factor of positive parenting interactions with children. Figure 1 shows the path diagram of the confirmatory factor analysis with items loading on one of the four domains, and domains loading on the general factor.

All factor loadings exceeded the desired threshold of 0.40, and were statistically significant (*p* < 0.001), except item 5 loading on the affection domain, and item 6 loading on the teaching domain. The model’s overall goodness of fit indices were as follows: χ^2^ = 617.65 (*df* = 373, *N* = 203); relative chi-square (χ^2^/df) = 1.66; CFI = 0.970; TLI = 0.967; and RMSEA = 0.057 (90% CI:0.049 -0.065). These results suggest a good (χ^2^/df < 2, CFI > 0.95 and TLI > 0.95) or, at least, acceptable (RMSEA < 0.08) fit of the model.

### Spanish PICCOLO Scores by Child Age and Gender

Table 2 shows the mean and standard deviation for the Spanish PICCOLO scores according to age of the child. Pearson’s correlation coefficients between child age (in months) and Spanish PICCOLO scores were computed. Statistically significant correlations were found between child age and scores for the teaching domain (*r* = 0.28; *p* < 0.001) and, to a lesser extent, between child age and total PICCOLO scores (*r* = 0.15, *p* = 0.033), indicating that teaching and positive parenting interactions were higher with older children, as also found in the original PICCOLO sample (Roggman et al., 2013b) and in a Turkish sample (Bayoğlu et al., 2013). In the Turkish sample, a statistically significant negative correlation was also found between child age and scores for the affection domain (*r* = -0.32, *p* < 0.001), which means that affection was higher with younger children. However, in the Spanish sample, the obtained negative correlation between those variables was not significant (*r* = -0.06, *p* = 0.431).

**Table 2:** Spanish PICCOLO scores by child age.

		Aff		Resp		Enc		Teach		Total	
		(6 items)		(7 items)		(6 items)		(7 items)		(26 items)	
Child age	*N*	*M*	*SD*	*M*	*SD*	*M*	*SD*	*M*	*SD*	*M*	*SD*
1 year	77	11.51	1.86	12.11	2.32	10.85	2.63	10.53	3.34	45.09	8.40
2 years	74	11.10	2.41	12.21	2.44	11.12	3.07	11.79	3.27	46.24	9.27
3 years	46	11.10	1.72	12.19	2.00	11.08	2.62	12.06	3.08	46.45	7.01

*Aff, Affection domain; Resp, Responsiveness domain; Enc, Encouragement domain; Teach, eaching domain; Total, otal score on the Spanish PICCOLO*.

With respect to child gender, the Student’s *t* test for independent samples found no statistically significant differences between boys (*n* = 114) and girls (*n* = 89) either for each domain or for the mean total PICCOLO scores. This result shows that, as expected based on previous studies (Bayoğlu et al., 2013; Roggman et al., 2013b), the positive parenting interactions observed in the Spanish mothers were not associated with child gender.

### Correlations Among PICCOLO Domains

Pearson’s correlation coefficients among Spanish PICCOLO domains were obtained. As shown in Table 3, the lowest correlation coefficient was found between the affection and teaching domains (*r* = 0.30), and the highest between responsiveness and encouragement (*r* = 0.66). Therefore, the PICCOLO domains were moderately to highly correlated with one another, although not at a level that would suggest that they measure the same construct. Indeed, all of the correlation coefficients between two different domains met the recommended criterion for discriminant validity, which requires a correlation between two constructs of less than 0.85 (Kline, 2015).

**Table 3:** Pearson’s correlation coefficients among Spanish PICCOLO domains (*N* = 203).

Domain	Affection	Responsive ness	Encourage ment	Teaching
Affection	1.0			
Responsiveness	0.53^∗∗^	1.0		
Encouragement	0.50^∗∗^	0.66^∗∗^	1.0	
Teaching	0.30^∗∗^	0.55^∗∗^	0.56^∗∗^	1.00

^∗^*p < 0.01*.

### Concurrent Validity

For a subsample of 64 participants, Spanish PICCOLO domain and total scores were examined in relation to child cognitive, language and motor skills outcomes, measured using the Bayley scales of infant development (BSID-III). For this analysis, Pearson’s correlations between mothers’ PICCOLO scores and children’s BSID-III scores were computed.

As shown in Table 4, some significant positive correlations (*p* < 0.05) were found between PICCOLO scores and some Bayley subscale scores. In particular, child cognitive Bayley scores were positively associated with mothers’ PICCOLO encouragement scores. These results indicated that the more a mother demonstrates encouragement behaviors with her child, the higher the child’s cognitive level.

**Table 4:** Pearson’s correlations between PICCOLO scores and children’s BSID-III scores (*N* = 64).

	BSID-III outcome					
	Cognitive		Language		Motor skill	
PICCOLO score	*r*	(*p*)	*r*	(*p*)	*r*	(*p*)
Affection	0.028	(0.826)	0.149	(0.239)	0.047	(0.747)
Responsiveness	0.225	(0.076)	0.320	(0.010)^∗^	0.152	(0.293)
Encouragement	0.276	(0.030)^∗^	0.294	(0.019)^∗^	0.016	(0.913)
Teaching	-0.035	(0.784)	0.281	(0.024)^∗^	-0.070	(0.628)
Total	0.146	(0.259)	0.325	(0.009)^∗^	-0.035	(0.812)

^∗^*p < 0.05, ^∗^*p* < 0.01*.

Child language Bayley scores were also positively associated with mothers’ PICCOLO responsiveness, encouragement, teaching, and total scores. That is, the higher a mother’s parenting scores (except for the affection domain), the higher the child’s language level. However, children’s motor skills were not associated with any of the mothers’ positive parenting domains.

## Discussion

The purpose of this study was to test the psychometric properties of the PICCOLO, an observational measure of parenting interactions with children that was developed in the United States (Roggman et al., 2013a), for use in practice and research on the Spanish population. The reliability and validity of the instrument was explored using a large sample of Spanish mothers and children aged from 10 to 47 months. The mean scores in all domains were similar between 1, 2 and 3 years of age, as shown in Table 2, with Responsiveness being the most constant dimension among ages. Affection decreased slightly from 1 to 2 years, and Encouragement showed a slight increase between the same ages. Teaching was the most variable dimension, increasing from 1 to 2 years and from 2 to 3. This could be interpreted as showing a mother’s tendency to adjust to the child’s interests and needs at all ages, a slight tendency to show more affection to younger children and to promote autonomy in older children, and a progressive increase in teaching behaviors with child’s age.

The analyses of the 29 original PICCOLO items showed that two items should be eliminated due to discrimination indices below the recommended limits. However, in item analysis it is important to keep in mind the main purpose of the instrument. The PICCOLO was designed as a useful parenting measure that predicts positive child outcomes. Indeed, the items contained in the PICCOLO were selected because they represented positive parenting interactions that research and theory suggested were related to children’s development (i.e., the items are causal indicators of children’s outcomes). As Bradley (2015) pointed out, using procedures that are standard practice in constructing scales (e.g., dropping items with low item-to-total correlations or weak factor loadings) can be a mistake for measures composed of causal indicators. Accordingly, we decided to include all of the original PICCOLO items for the Spanish version in order to maximize content validity and potential criterion-related validity, at the expense of a lower reliability of the measures.

Our findings showed good inter-rater reliability and internal consistency reliability of the Spanish PICCOLO domain and total scores, with similar values to those found in samples from Turkey (Bayoğlu et al., 2013) and the United States (Roggman et al., 2013a). Other observational measures of parenting that require complex coding or rating scales often require substantial training time for observers to accurately use the measures (Fuligni and Brooks-Gunn, 2013). In contrast, PICCOLO is a checklist of observable behaviors that can be learned relatively quickly to achieve accurate ratings of parenting interactions with children.

In relation to the dimensionality of the instrument, the results of the confirmatory factor analysis demonstrated that the instrument has a four-factor structure of first order domains that collapses into a single, second-order factor of parenting. The four first-order factors corresponded to the theoretical constructs (affection, responsiveness, encouragement, and teaching), which the original authors hypothesized in developing the instrument (Roggman et al., 2013a). Indeed, the original authors recommend scoring all four domains of the PICCOLO with families “in order to be (a) *Positive*: Most parents have strengths in more than one of the four domains, (b) *Practical*: Practitioners can see more parenting strengths if they are watching for all four domains, (c) *Culturally sensitive*: Different cultures emphasize different domains, and (d) *Sensible*: Using all four domains helps practitioners see the whole picture” (Roggman et al., 2013a, p. 23). Furthermore, the fact that these four domains collapse into a general second-order factor also justifies the use of a total score of positive parenting interactions in the evaluation. The fit of the model, for the confirmatory factor analysis, was acceptable, which implies construct validation evidence for the Spanish version of the PICCOLO.

Further construct validation evidence was compiled by examining the association between the PICCOLO scores and expected parenting-related constructs, such as child age and child gender. In this study, teaching domain and total PICCOLO scores were positively correlated with child age, as also found in the original PICCOLO sample (Roggman et al., 2013b) and the Turkish sample (Bayoğlu et al., 2013). In the Turkish sample, a statistically significant negative correlation was also found between child age and scores for the affection domain, which means that affection was higher with younger children. However, in the Spanish sample, the correlation between those variables was not significant. These differences may reflect different cultural values about parenting or about the parents’ goals for their children’s developmental outcomes (Tuttle et al., 2012). With respect to the relationship between positive parenting interactions and child gender, our results showed no statistically significant differences between boys and girls for the mean PICCOLO scores, as expected based on previous studies (Bayoğlu et al., 2013; Roggman et al., 2013a).

Concurrent validity between the PICCOLO and the Bayley scales, assessed using a subsample of 64 participants from the general validation sample, showed that children’s cognitive development was positively associated with mothers’ PICCOLO score in encouragement. These results are consistent with those of Hubbs-Tait et al. (2002); Bernier et al. (2010, 2012), and Fay-Stammbach et al. (2014). On the other hand, the children’s language Bayley scores were positively associated with the mothers’ PICCOLO responsiveness, encouragement, teaching, and the total scores. Our results are consistent with those of Hirsh-Pasek and Burchinal (2006) and Tamis-LeMonda et al. (2001, 2014). As expected, most of the very clear relations established in previous studies were found: linguistic development was strongly associated with responsiveness and teaching, and also encouragement; cognitive development was associated with encouragement, but not teaching, as expected. As also expected, parenting, as assessed using the PICCOLO, was not associated with motor skills. The literature about the parental behaviors assessed using the PICCOLO during play situations, such as book-reading, symbolic play, block constructions and so on, does not report any associations between these kinds of parental behaviors and motor skills.

Affection was not associated with any Bayley measure. This was not a totally expected result because, although the literature reports more conclusive data about the relationships between affection and social emotional development (Laible et al., 2000; Zhou et al., 2002; Caspi et al., 2004; Sanders et al., 2004), affection is especially relevant for the development of a secure attachment, and this in turn constitutes a good basis for general development (Mikulincer and Shaver, 2007; Thompson, 2008). Nevertheless, a larger sample of participants is needed to confirm these results.

It would also be interesting to explore predictive validity, correlating parenting measures with later measures of child development at time 2, as done by Roggman et al. (2013a,b). The authors found, among other results, that PICCOLO total and domain scores at ages 1 and 2 years were significantly associated with children’s cognitive development measured using the Bayley scales at age 3 years, and with linguistic development assessed at ages 3 and 5 using the Peabody Picture Vocabulary Test-III (Dunn and Dunn, 1997). In future studies, this aspect will be analyzed in a sample of about 100 normally developing children aged between 18 and 30 months, evaluating parenting at time 1 and relating this variable to developmental outcomes, assessed using the Bayley Scales, as already done in a small sample (Rivero et al., 2017).

This study has some limitations. First, our participants were all mothers; thus, our results might not be generalizable to other caregivers such as fathers or grandparents. In future studies the PICCOLO should be validated in other populations since gender differences in parental behaviors have been established (Cabrera et al., 2017). Secondly, our results come from observing mother-child interactions at home, so other settings where teacher interactions with young children take place should be tested as well (Jump Norman and Christiansen, 2013).

Furthermore, this study was conducted in families mostly belonging to the middle and upper middle classes, with a monthly family income and study level both higher than the average of the Spanish population. In comparison with the other published studies on validation of the PICCOLO -the original study from the United States and one from Turkey- our Spanish sample scored higher in all dimensions and at all ages, with the only exception of Affection in Turkey when children were 1 and 2 years of age. This could be explained by the higher level of maternal education in our sample, compared to the United States (Roggman et al., 2013b) and Turkey (Bayoğlu et al., 2013). A higher maternal educational level has been associated with parental practices, as mothers with a higher educational level show more parental behaviors that promote their children’s development (Bornstein et al., 2007; Brophy-Herb et al., 2012). Having a low income or limited education may certainly affect parenting behaviors (Roubinov and Boyce, 2017). The Brazilian-Portuguese validation is not comparable, since the sample included only children aged 18 months old.

Finally, although this study specifically concerned validation of the scale within a sample from Spain, replication of the validation and reliability procedures in other Spanish-speaking samples could extend the utility of this scale. We expect that the Spanish PICCOLO would be useful in other countries with substantial Spanish speaking populations.

## Conclusion

For infants and toddlers, a responsive home environment that includes good parenting and positive parent-child interactions is important for child development. The Spanish version of the PICCOLO, an observational measure of parent-child interactions for infants and toddlers, meets the criteria for a reliable and valid measurement instrument of mothers’ parenting interactions with children aged from 10 to 47 months. The psychometric properties of the Spanish version of the PICCOLO make it appropriate for general research purposes but also for program evaluation of home-visiting and other parenting-support interventions in Spain. For early intervention professionals in Spain, an observational measure such as the PICCOLO may be very useful and can help to better establish and expand a family-centered approach in Early Interventions programs (Mas et al., 2016; Vilaseca et al., 2017). Following a collaborative model of working together with parents, it is possible to identify what parents are already doing well to support their children’s development through their daily routines (McWilliam, 2010, 2016) and practitioners can build on those strengths by helping parents to increase support for children’s development at home, both in those children at-risk for environmental reasons or in children with established disabilities.

## Data Availability

The datasets for this study are not made publicly available because parents have allowed to our research team to video record their children, but not to publish any data or image.

## Author Contributions

All authors RV, MR, RB, EN-P, MJC, FF, CVV, MI, and LR made substantial contributions to conception and design, and/or acquisition of data, and/or analysis and interpretation of data; and participated in drafting the article or revising it critically for important intellectual content; and gave final approval of the version to be submitted.

## Conflict of Interest Statement

The authors declare that the research was conducted in the absence of any commercial or financial relationships that could be construed as a potential conflict of interest.
